# Innovative Agents in Multiple Myeloma

**Published:** 2014-05-01

**Authors:** Beth Faiman, Tiffany Richards

**Affiliations:** From Cleveland Clinic Foundation, Cleveland, Ohio, and University of Texas MD Anderson Cancer Center, Houston, Texas

## Abstract

Multiple myeloma (MM) remains an incurable cancer of the bone marrow plasma cells. However, the overall survival of patients with MM has increased dramatically within the past decade. This is due, in part, to newer agents such as immunomodulatory drugs (lenalidomide, thalidomide, and pomalidomide) and proteasome inhibitors (bortezomib, carfilzomib, MLN9708). These and several other new classes of drugs have arisen from an improved understanding of the complex environment in which genetic changes occur. Improved understanding of genetic events will enable clinicians to better stratify risk before and during therapy, tailor treatment, and test the value of personalized interventions. The ultimate goal in this incurable disease setting is to reduce the impact of cancer- or chemotherapy-related side effects. Nurses and advanced practitioners are integral to the treatment team. Thus, each should be aware of changes to the current drug landscape. Targeted drugs with sophisticated mechanisms of action are currently under investigation. Patients gain access to newer drugs within the context of clinical trials. Awareness of such trials will help accrual and determine if therapeutic benefit exists. In this article, we will describe new agents with unique and targeted mechanisms of action that have activity in patients with relapsed and/or refractory multiple myeloma.

Multiple myeloma (MM) is a rare cancer of the bone marrow plasma cells that affects approximately 70,000 individuals in the United States (Howlader, Noone, Krapcho, Neyman, & Kroenen, 2014). Symptoms of MM include bone damage, anemia, and/or renal insufficiency in the presence of a monoclonal protein. The etiology of MM is unknown. Through a series of genetic changes and mutational alterations, the clonal plasma cell will evolve (Faiman & Bilotti, 2013). Most patients will ultimately become resistant to treatment. Although this condition is still considered incurable, the survival of patients with MM has increased in part due to the use of drugs that differ from traditional chemotherapy in their unique mechanisms of action. Many of the newer agents have arisen from an improved genomic understanding of MM development, chromosomal changes, and the bone marrow microenvironment (Fonseca & Monge, 2013). See the Figure below for a visual representation of the bone marrow microenvironment.

**Figure 1:**
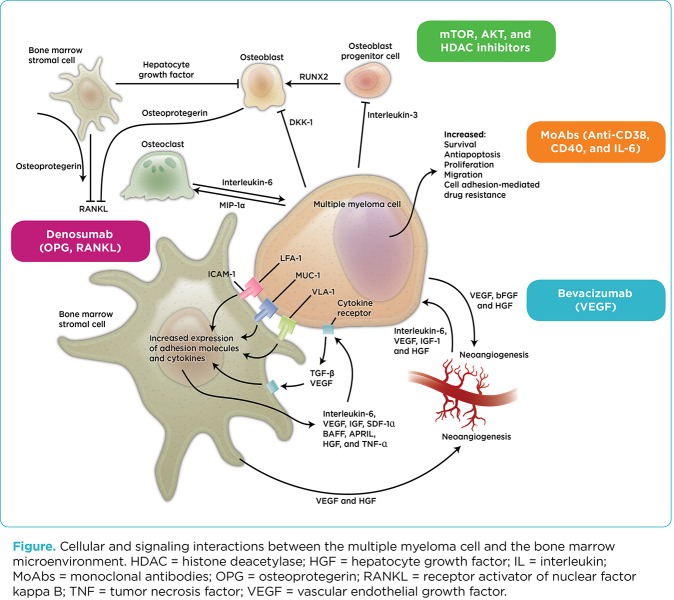
Cellular and signaling interactions between the multiple myeloma cell and the bone marrow microenvironment. HDAC = histone deacetylase; HGF = hepatocyte growth factor; IL = interleukin; MoAbs = monoclonal antibodies; OPG = osteoprotegerin; RANKL = receptor activator of nuclear factor kappa B; TNF = tumor necrosis factor; VEGF = vascular endothelial growth factor.

Within the past decade, patients with MM have begun to live longer than ever (Kastritis et al., 2010; National Comprehensive Cancer Network [NCCN], 2013; Reece et al., 2009; Richardson et al., 2010). In one review, the median survival of patients diagnosed prior to 1997 was nearly 2.5 years compared with nearly 4 years for patients diagnosed in the decade after that (Kumar et al., 2008). However, patients with MM refractory to both immunomodulatory drugs (IMiDs) and bortezomib (Velcade) have a particularly poor prognosis (Kumar et al., 2012). Patients eventually develop refractory disease, which leads to a need for newer drugs with innovative mechanisms of action. Several drugs have demonstrated activity against relapsed MM, but the optimal dosing, schedule, and drug combination require further investigation in randomized controlled trials.

## Monoclonal Antibodies

Monoclonal antibody (MoAb)-directed therapies are often used in hematologic malignancies such as chronic lymphocytic leukemia and non-Hodgkin lymphoma. Several antibodies with various antigen or bone marrow targets have been investigated in patients with MM during the past decade (Lonial et al., 2013; Tai & Anderson, 2011). The MoAbs have multiple mechanisms of action, including cellular and complement toxicity as well as the targeting of proteins, growth factors, and their receptors. The benefits of these drugs have been well described in lymphomas and other cancers. Research efforts attempting to gain insight into effective MoAb therapy in MM continue.

**Table 1:**
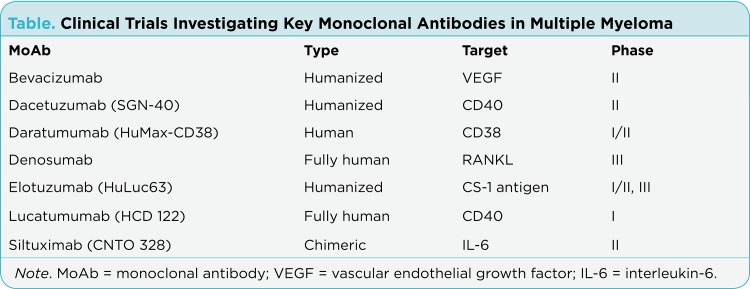
Clinical Trials Investigating Key Monoclonal Antibodies in Multiple Myeloma

Types of MoAbs are derived from murine (mouse), chimeric (using mouse variable regions and grafting into human constant regions), human (transferring human immunoglobulin genes into the mouse genome), and humanized (grafting murine into human antibodies) cells. Several agents with interesting therapeutic targets provide new treatment options for patients. Similar to existing MoAbs, these drugs seem to be most effective when given in combination with other agents. Key MoAbs in clinical trials include elotuzumab (HuLuc63), dacetuzumab (SGN-40), siltuximab (CNTO 328), daratumumab (HuMax-CD38), bevacizumab (Avastin), and denosumab (Xgeva); see the Table above for more information.

**Elotuzumab**

Elotuzumab is a fully humanized MoAb that targets the antigen CS-1. It is highly expressed by 97% of patients with MM (Hsi et al., 2008; Tai et al., 2009). CS-1 mediates tumor cell adhesion and supports tumor growth via interaction with bone marrow stromal cells. Elotuzumab is believed to act on natural killer (NK) cell-mediated antibody-dependent cellular cytotoxicity (ADCC), a major mechanism of cell death that may be compromised due to underlying immune dysfunction from the MM disease itself.

In a phase II study of patients with relapsed/refractory MM, the combination of elotuzumab, lenalidomide (Revlimid), and low doses of dexamethasone produced an overall response rate of 92% at a dose of 10 mg/kg of elotuzumab at 18 months of therapy (Lonial et al., 2013). Toxicities were mild and manageable. The most frequently encountered grade 3 or 4 toxicities were neutropenia (21%), anemia (13%), and fatigue (8%).

**Dacetuzumab**

Dacetuzumab is a humanized anti-CD40 MoAb that works by downregulating interleukin-6 (IL-6) receptor, making cells unresponsive to IL-6 stimulation, and inducing cell death in MM cell lines (Tai et al., 2005). Recent studies suggest that CD40—a transmembrane protein and part of the tumor necrosis factor (TNF) "superfamily"—plays an essential role in mediating immune and inflammatory responses. These include memory B-cell development, T-cell–dependent immunoglobulin class switching, and germinal center formation (Bolduc et al., 2010).

**Lucatumumab**

Lucatumumab is a fully human anti-CD40 MoAb that inhibits MM cell growth. Results of a phase I study reported that lucatumumab is well tolerated up to 4 to 5 mg/kg. Modest clinical activity was observed in patients with relapsed/refractory MM. The efficacy of lucatumumab as a single agent and/or in combination with other drugs remains to be seen, but with a tolerable side-effect profile, future studies are planned (Bensinger et al., 2012).

**Siltuximab**

Siltuximab is an anti–IL-6 chimeric MoAb. Interleukin-6 is a proinflammatory cytokine and a major growth factor for MM cells (Voorhees et al., 2013a). In one trial, patients were randomized to receive siltuximab and bortezomib or placebo and bortezomib. While the results showed a 13% improved progression-free survival (PFS) rate, this was not statistically significant. In fact, overall survival was 937 days among patients who received siltuximab and bortezomib vs. 1,121 days for those who received bortezomib and placebo (*(p* = .103). Unfortunately, the combination of siltuximab and bortezomib may not represent a promising new treatment for MM, but future combinations of siltuximab with other agents may elicit better results (Orlowski et al., 2012).

**Daratumumab**

Daratumumab is a CD38 MoAb that has shown promising preliminary efficacy in a phase I/II study. CD38 is a transmembrane glycoprotein responsible for receptor-mediated adhesion, signal transduction, and regulation of intracellular calcium (de Weers et al., 2011; van der Veer et al., 2011). Multiple myeloma cells express relatively high levels of CD38, thus representing a target for drug therapy. Daratumumab is an exciting MoAb in that it eliminates tumor cells expressing the CD38 antigen. de Weers and colleagues (2011) hypothesized that daratumumab may carry out its function through ADCC, complement-dependent cytotoxicity (CDC), and apoptosis.

Daratumumab has been studied as a single agent and in combination with lenalidomide and dexamethasone (Plesner et al., 2013; Lokhorst et al., 2013). In a phase I/II dose-escalation study, doses of single-agent daratumumab > 4 mg/kg led to unprecedented results. A significant reduction in serum paraproteins and bone marrow plasmacytosis in heavily pretreated patients with MM was noted (Lokhorst et al., 2013). In a second phase I/II study, 6 patients with relapsed and refractory MM received lenalidomide and dexamethasone with varying doses of daratumumab, ranging from 2 to 16 mg/kg for 8 weeks, then twice a month for 16 weeks, and once monthly until disease progression. A decline in serum M protein and bone marrow plasma cells was observed in all patients, with 50% achieving an impressive very good partial response or better (Plesner et al., 2013). According to the investigators, the toxicity of daratumumab was minimal, with myelosuppression cited as the most common side effect.

**Bevacizumab**

Bevacizumab is an antibody that attacks the vascular endothelial growth factor (VEGF) antigen and is approved by the US Food and Drug Administration (FDA) for the treatment of colorectal cancer and glioblastoma. VEGF is upregulated in MM, and increased levels may theoretically correspond to increased MM activity (Hideshima, Chauhan, & Anderson, 2004). Bevacizumab has been studied in several trials in combination with either bortezomib, lenalidomide, or thalidomide (Thalomid) with low-dose dexamethasone (Callander et al., 2009; Somlo et al., 2011). Grade 3 fatigue is the most commonly cited side effect in the trials, but few patients have achieved greater than a partial response to bevacizumab.

In a phase II multicenter study of patients with relapsed/refractory MM, patients were randomized to receive bortezomib (1.3 mg/m^2^ on days 1, 4, 8, and 11 of each 21-day cycle) and either placebo or bevacizumab (15 mg/kg on day 1 of each cycle) for up to 8 cycles. Patients who responded to bevacizumab plus bortezomib continued until disease progression. The primary endpoint was PFS. The median PFS was 6.2 months (95% confidence interval [CI], 4.4–8.5 months) in patients who received bortezomib and bevacizumab and 5.1 months in patients who received bortezomib and placebo (95% CI, 4.2–7.2 months; White et al., 2012). Although the MoAb was well tolerated, the results were nonsignificant. However, it is important to note that no new safety concerns were identified. Additional bevacizumab-based studies that bear promise to identify the optimal combination regimen are ongoing.

**Denosumab**

Denosumab is a high-affinity MoAb approved for the treatment of osteoporosis in postmenopausal women and for the prevention of skeletal-related events (SREs) in patients with bone metastases from solid tumors (Henry et al., 2011). Denosumab works most notably as a receptor activator of nuclear factor êB ligand (RANKL) inhibitor. Stimulation of osteoclasts (responsible for bone breakdown) and osteoblasts (responsible for bone repair) is controlled in part by the equilibrium between the OPG (osteoprotegerin), RANKL, and RANK triad. RANKL binds to the RANK receptor found on the surface to osteoclasts. Binding of RANKL results in stimulation of osteoclast activity and leads to bone breakdown. OPG is a "decoy receptor" produced by osteoblasts. OPG inhibits osteoclastogenesis by binding to RANKL, which inactivates RANKL. RANKL is the key mediator of osteoclastogenesis, thus administering MoAbs such as denosumab blocks RANKL activity (Henry et al, 2011; Raje et al., 2013).

In the phase III registration trial, denosumab was noninferior to zoledronic acid in preventing or delaying the first on-study SREs in patients with advanced cancer metastatic to bone or myeloma. However, MM patients comprised only 10% of total patients (93 in the denosumab arm and 87 in the zoledronic acid arm), and noninferiority could not be concluded based on small numbers of MM patients. It is important to note that the incidence of osteonecrosis of the jaw with prolonged exposure to denosumab increases after 1 year of therapy, similar to other agents used to decrease SREs such as zoledronic acid (Lipton et al., 2013). A subgroup analysis of MM patients in 2013 confirmed that they were underrepresented and therefore the use of denosumab should be limited in patients with MM until data from a larger trial are available (Raje et al., 2013).

A phase III trial of denosumab compared with zoledronic acid in the treatment of bone disease in patients with MM (ClinicalTrials.gov identifier NCT01345019) is actively recruiting bisphosphonate-naive MM patients. The study’s primary purpose is to determine whether denosumab is noninferior to zoledronic acid in the treatment of bone disease from MM.

## Histone Deacetylase Inhibitors

**Vorinostat**

Vorinostat (Zolinza) is an oral histone deacetylase (HDAC) inhibitor currently approved by the FDA for treatment of cutaneous T-cell lymphoma. Histones regulate genes and proteins integral for tumor growth and survival. In the phase III global VANTAGE 088 study, 637 patients with relapsed and/or refractory MM were randomized in a 1:1 fashion to receive 21-day cycles of bortezomib at a dose of 1.3 mg/m^2^ IV on days 1, 4, 8, and 11 in combination with oral vorinostat 400 mg/d, or matching placebo, on days 1 to 14. The primary endpoint for this trial was PFS (Dimopoulos et al., 2011).

The results of the study showed that patients treated with vorinostat and bortezomib had a 23% reduction in the risk of disease progression compared to bortezomib alone (hazard ratio of 0.774; *p* = .01) and a median PFS of 7.6 months in the vorinostat plus bortezomib arm vs. 6.8 months in the bortezomib arm (Dimopoulos et al., 2011).

In the international, multicenter, open-label study of vorinostat in combination with bortezomib in heavily pretreated, double-refractory patients (those who have failed to respond to treatment with bortezomib and an immunomodulatory agent) with relapsed MM (VANTAGE 095), the combination of vorinostat plus bortezomib had an overall response rate (ORR) of 17% and a clinical benefit rate (CBR) of 31% as assessed by International Myeloma Working Group (IMWG) criteria (Siegel et al., 2010).

In a phase I study, researchers combined vorinostat with lenalidomide and dexamethasone in 32 patients with relapsed/refractory myeloma (Siegel et al., 2014). The ORR for all patients was 47%, with a median time to response of 91 days. In patients who were refractory to lenalidomide, the ORR was 10%, with 40% having stable disease. In patients who were refractory to proteasome inhibitors, the ORR was similar to that for those who were resistant to lenalidomide, with 15% of patients achieving greater than a partial response (PR) and 39% having stable disease. The main adverse events reported included anemia, thrombocytopenia, diarrhea, fatigue, and cough (Siegel et al., 2014).

**Panobinostat**

Panobinostat (LBH-589) is a nonselective HDAC inhibitor that has demonstrated activity in MM. PANORAMA 2 is a single-arm, phase II study of panobinostat plus bortezomib plus dexamethasone in patients with MM who are refractory to bortezomib therapy and have received two or more prior lines of therapy (Richardson et al., 2013). Patients were treated in two phases. In the first treatment phase, patients received eight 3-week cycles of oral panobinostat (20 mg on days 1, 3, 5, 8, 10, and 12), IV bortezomib (1.3 mg/m^2^ on days 1, 4, 8, and 11), and oral dexamethasone (20 mg on the day of and the day after bortezomib). If a patient demonstrated stable disease, the individual proceeded to phase II. This treatment phase consisted of four 6-week cycles of panobinostat (20 mg three times a week for 2 weeks on, 1 week off, and repeat) with bortezomib (1.3 mg/m^2^ on days 1, 8, 22, and 29) and dexamethasone (20 mg on the day of and the day after bortezomib). Side effects of therapy were manageable.

One patient (1.8%) achieved a near complete response, with 18 patients achieving a PR (32.7%) and an additional 10 patients (18.2%) achieving a minimal response. The median duration of response was 6 months, with a PFS of 5.4 months. In those patients with high-risk cytogenetics (del 17p, t[4:14], t[14:16]), the ORR was 42.9%; the median duration of response in these patients was not reported (Richardson et al., 2013).

A phase I/II study of panobinostat combined with melphalan in 40 patients with relapsed/refractory myeloma found a maximum tolerated dose (MTD) of 20 mg of panobinostat with melphalan 0.05 mg/kg on days 1, 3, and 5 of a 28-day cycle (Berenson et al., 2013). An ORR of 7.5% was observed, with 57.5% achieving stable disease. The main > grade 3 adverse events reported included thrombocytopenia (33.9%), neutropenia (30.5%), lymphopenia (22.5%), anemia (15%), hyponatremia (7.5%), hypophosphatemia (5%), increased creatinine (2.5%), hypocalcemia (2.5%), hyperkalemia (2.5%), fatigue (2.5%), rash (2.5%), and deep-vein thrombosis (2.5%).

In an attempt to build on the synergy between HDAC inhibitors and proteasome inhibitors, researchers combined panobinostat with carfilzomib (Kyprolis) in patients who had relapsed after at least one prior line of therapy (Berdeja et al., 2013). A total of 34 patients were enrolled; the ORR was 64% for all cohorts and 67% in patients with bor- tezomib-refractory disease. The most common side effects were myelosuppression (> grade 3, 45%), fatigue (> grade 3, 11%), and nausea/vomiting (> grade 3, 9%). These trials demonstrate panobinostat as a promising treatment for relapsed MM in combination with bortezomib and potentially other novel agents (Richardson et al., 2011).

## mTOR Inhibitors

The mammalian target of rapamycin (mTOR) is a protein responsible for a variety of activities within the cell. mTOR inhibitors work by affecting tumor cell growth, including growth, proliferation, survival, transcription, and protein synthesis. Thus, mTOR has become a popular target for new therapeutics to inhibit tumor cell growth (Hoang et al., 2010). Two main types of mTORs exist (mTOR1 and mTOR2), each responsible for different activities. The main function of mTOR1 is controlling protein synthesis; it is activated by insulin, growth factors, and oxidative stress. mTOR2 is mainly responsible for regulating the cytoskeleton; it is activated by insulin, growth factors, and nutrient levels (Hoang et al., 2010; 2012). Two mTOR inhibitors are currently undergoing clinical trials in patients with multiple myeloma: temsirolimus and everolimus.

Preclinical work suggested the mTOR inhibitor rapamycin to be potentially effective in combination with lenalidomide (then known as CC-5013; Raje et al., 2004). In a phase I study of temsirolimus in 16 patients with relapsed and refractory MM, 1 (6%) partial response and 5 (31%) minor responses were observed. The synergy observed in in vitro studies led researchers to study the combination of other mTOR inhibitors with other novel agents.

**Temsirolimus and Everolimus**

Investigators conducted phase I studies of lenalidomide and the mTOR inhibitor temsirolimus (CCI-779). In a study by Hofmeister and colleagues (2011), 21 patients with relapsing myeloma were assigned to receive lenalidomide and temsirolimus in combination. Of the 19 patients evaluable for response, 2 (11%) obtained a partial remission, 6 (31%) had a marginal response, and 15 (78%) had stable disease. Of the 21 patients on the study, 4 had received prior lenalidomide therapy; however, only 1 patient had progressed on lenalidomide. The adverse effects observed with the regimen included fatigue, neutropenia ( grade 3, 33%), anemia ( grade 3, 10%) hypophosphatemia (76%, grade 3, 57%), hypokalemia (62%, grade 3, 29%), rash (76%), and infection (23%; Hofmeister et al., 2011).

Ghobrial et al. (2011) studied the combination of bortezomib and temsirolimus in a phase I/II study of 63 patients with relapsed and refractory myeloma. Of the 20 patients enrolled in the phase I portion of the study, 10% achieved PR and 20% achieved minimal response (MR). In the phase II study, 33% achieved PR, with 47% obtaining MR. Of the 33 patients refractory to bortezomib, a total of 11% in both studies obtained PR and 21% obtained MR. The main toxicities reported included thrombocytopenia, neutropenia, anemia, fatigue, GI toxicity, and peripheral neuropathy. The results of this study demonstrate that bortezomib plus temsirolimus is an active regimen, and that the two agents may overcome bortezomib resistance when used in combination (Ghobrial et al., 2011).

In a phase I study, 26 patients with relapsed and refractory myeloma received everolimus in combination with lenalidomide for 21 days of a 28-day cycle (Yee et al., 2011). The MTD was defined as 15 mg of lenalidomide and 5 mg of everolimus. Of the 19 patients who completed 2 cycles of therapy, the response rate of PR was 21%, with 37% achieving a MR.

## Akt Inhibitors

Akt protein kinase B is an enzyme that plays an important role in glucose metabolism, cell survival, and transcription. Akt is required for the metabolism of glucose and for the transportation of glucose across cell membranes. Akt also plays a role in cell survival and is thought to promote cell survival in mutated cells as well as the initiation and progression of malignancies. The main role of Akt is to block the phosphatidylinositol-3 kinase (PI3K) pathway (Ghobrial et al., 2011; Huston et al., 2008).

Akt inhibitors demonstrate some promise particularly when combined with bortezomib. Phase II and III trials are ongoing to determine if the combination of Akt inhibitors with other novel agents is superior to other drug regimens. The toxicity profile is manageable, with hematologic effects, GI toxicities, and fatigue as the most common adverse events thus far. Combination studies are ongoing.

**Afuresertib**

A phase I/II study of afuresertib, bortezomib, and dexamethasone was conducted among 67 patients with relapsed/refractory myeloma (Voorhees et al., 2013b). The MTD was defined as afuresertib 150 mg; bortezomib 1.3 mg/m^2^ on days 1, 4, 8, and 11; and dexamethasone 40 mg on the days of bortezomib. The ORR was 41%, with an additional three patients achieving a minimal response. In this study, the majority of patients had received prior proteasome inhibitor and immunomodulatory agents; however, it is not known how many of these patients were resistant to therapy. The main dose-limiting toxicities included elevation of liver function studies (1/6), erythema multiforme (1/6), rash (1/6), diarrhea (1/6), and thrombocytopenia (1/6). Other adverse events reported were fatigue (51%), thrombocytopenia (39%), nausea (39%, dyspepsia (36%), constipation (36%), hyperglycemia (30%), vomiting (28%), anemia (25%), and dizziness (21%).

**Perifosine**

Richardson et al. (2011) conducted a phase I/II study of perifosine, a drug that has been researched and has failed in other cancers, in combination with bortezomib with or without dexamethasone among 84 patients with relapsed and refractory myeloma. Of the 73 patients evaluable for response, 22% achieved PR and 19% achieved a MR, with a median PFS of 6.4 months among all responders. Of the 53 patients who were refractory to prior bortezomib therapy, 13% achieved PR and 19% MR, with a median PFS of 5.7 months. While the response rates are small, it is encouraging that 13% of bortezomib-refractory patients responded to the combination (Richardson et al., 2011).

In a phase III randomized controlled study, 135 patients received either perifosine, bortezomib, and dexamethasone or placebo, bortezomib, and dexamethasone (Richardson et al., 2013). Responses were similar between the two arms, with 20.3% of patients responding in the perifosine arm and 27.3% responding in the placebo arm. Overall survival was not statistically significant between perifosine and placebo (141.9 vs. 83.3 weeks; *(p* = .356).

In another phase I trial by Jakubowiak and colleagues (2012), 31 patients with relapsed and refractory myeloma were given escalating doses of perifosine, lenalidomide, and dexamethasone. Of the 30 evaluable patients, 2 had prior lenalidomide exposure but were not refractory to lenalidomide, and 59% were refractory to thalidomide. The ORR for those achieving PR was 50% and 23% achieved a MR. The median duration of response was 9.2 months for all patients. In those patients who were refractory to thalidomide, the response rate of those achieving greater than PR was 23%. The most common grade 1 and 2 nonhematologic adverse effects reported included fatigue (48%), diarrhea (45%), hyperglycemia (32%), and nausea (32%). The main grade 3 and 4 toxicities included neutropenia (25%), hypophosphatemia (23%), thrombocytopenia (16%), leukopenia (13%), arthralgia (10%), and hyperglycemia (10%). While this regimen did demonstrate response rates of > 50%, it is difficult to ascertain if the response rates observed are from the combination of lenalidomide and dexamethasone rather than from the addition of perifosine to the regimen.

## Conclusion

Average survival for individuals with MM has increased, yet most patients experience periods of remission and relapse. Unfortunately the MM eventually becomes refractory to treatment. Thus, novel agents with sophisticated mechanisms of action are particularly exciting to explore. It is helpful to understand the underlying biology and pathways of myeloma cell development in assessing potential new therapies. Genetic expression and protein signaling pathways are promising areas of research. Newer drugs aim to target affected pathways to provide future treatment options for patients. l

## Disclosure

Ms. Faiman has served on speakers bureaus for Millennium, Onyx Pharmaceuticals, and Celgene. Ms. Richards has acted as a consultant for Onyx Pharmaceuticals and Celgene.
